# Special AT‐rich sequence‐binding protein 2 (*Satb2*) synergizes with *Bmp9* and is essential for osteo/odontogenic differentiation of mouse incisor mesenchymal stem cells

**DOI:** 10.1111/cpr.13016

**Published:** 2021-03-04

**Authors:** Qiuman Chen, Liwen Zheng, Yuxin Zhang, Xia Huang, Feilong Wang, Shuang Li, Zhuohui Yang, Fang Liang, Jing Hu, Yucan Jiang, Yeming Li, Pengfei Zhou, Wenping Luo, Hongmei Zhang

**Affiliations:** ^1^ Chongqing Key Laboratory for Oral Diseases and Biomedical Sciences The Affiliated Hospital of Stomatology of Chongqing Medical University Chongqing China; ^2^ Department of Pediatric Dentistry The Affiliated Stomatology Hospital, Chongqing Medical University Chongqing China; ^3^ Chongqing Municipal Key Laboratory of Oral Biomedical Engineering of Higher Education Chongqing China

**Keywords:** *Bmp9*, bone, mesenchymal stem cells, odontogenic differentiation, osteo, *Satb2*, tooth

## Abstract

**Objectives:**

Mouse incisor mesenchymal stem cells (MSCs) have self‐renewal ability and osteo/odontogenic differentiation potential. However, the mechanism controlling the continuous self‐renewal and osteo/odontogenic differentiation of mouse incisor MSCs remains unclear. Special AT‐rich sequence‐binding protein 2 (SATB2) positively regulates craniofacial patterning, bone development and regeneration, whereas SATB2 deletion or mutation leads to craniomaxillofacial dysplasia and delayed tooth and root development, similar to bone morphogenetic protein (BMP) loss‐of‐function phenotypes. However, the detailed mechanism underlying the SATB2 role in odontogenic MSCs is poorly understood. The aim of this study was to investigate whether SATB2 can regulate self‐renewal and osteo/odontogenic differentiation of odontogenic MSCs.

**Materials and methods:**

*Satb2* expression was detected in the rapidly renewing mouse incisor mesenchyme by immunofluorescence staining, quantitative RT‐PCR and Western blot analysis. Ad‐*Satb2* and Ad‐si*Satb2* were constructed to evaluate the effect of *Satb2* on odontogenic MSCs self‐renewal and osteo/odontogenic differentiation properties and the potential role of *Satb2* with the osteogenic factor bone morphogenetic protein 9 (*Bmp* *9*) in vitro and in vivo.

**Results:**

*Satb2* was found to be expressed in mesenchymal cells and pre‐odontoblasts/odontoblasts. We further discovered that *Satb2* effectively enhances mouse incisor MSCs self‐renewal. *Satb2* acted synergistically with the potent osteogenic factor *Bmp9* in inducing osteo/odontogenic differentiation of mouse incisor MSCs in vitro and in vivo.

**Conclusions:**

*Satb2* promotes self‐renewal and osteo/odontogenic differentiation of mouse incisor MSCs. Thus, *Satb2* can cooperate with *Bmp9* as a new efficacious bio‐factor for osteogenic regeneration and tooth engineering.

## Graphical abstract

The graphical abstract showed that *Satb2* and *Bmp9* were expressed in mesenchymal stem cells of mouse incisor growth region. *Satb2* acted synergistically with the potent osteo/odontogenic factor *Bmp9* in mouse incisor MSCs. *Satb2* could as a new efficacious bio‐factor for osteogenic regeneration and tooth engineering in cooperation with *Bmp9*.

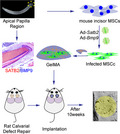

## 1 INTRODUCTION

Mesenchymal stem cells (MSCs) are subjected to complex and tight regulation by diverse growth factors and cytokines in osteogenic regeneration and tooth engineering.^1^, ^2^ The continuously growing mouse incisor provides a superior model for understanding the mechanisms of odontogenic MSCs self‐renewal and osteo/odontogenic differentiation.^3^ In the mouse incisor apical papilla, the pool of continuously growing MSCs (named mouse incisor MSCs) provides precursor cells to maintain renewal growth and mineralization.^4^, ^5^ Therefore, the identification and characterization of important regulatory factors that govern the stem cell properties and differentiation potential of mouse incisor MSCs should significantly expand our understanding of osteogenic regeneration and thus facilitate tooth engineering. Our previous studies indicated that mouse incisor MSCs, which are recognized as one of the most promising odontogenic stem cells in bone and tooth regeneration engineering, show the osteo/odontogenic differentiation potential would induced by effective biological factors.^6^, ^7^

As a member of a special family of AT sequence‐binding proteins, special AT‐rich sequence‐binding protein 2 (SATB2), which binds to nuclear matrix‐associated regions and activates gene transcription programs, is a multifunctional regulator involved in development, particularly in craniofacial patterning, palate formation, osteoblast differentiation and bone regeneration.^8^ Human *SATB2* is a highly evolutionarily conserved chromatin remodelling gene located on chromosome 2q33.1,^9^ and mutation of *SATB2* induces not only severe bone‐associated conditions, including cleft palate, facial cleft, micrognathia and alveolar bone dysplasia^10^, ^11^, ^12^, ^13^ but also odontogenic abnormalities, including missing teeth, delayed tooth and root development.^14^, ^15^, ^16^ *Satb2* is responsible for osteoblast function to promote osteoblastogenesis and enhance bone regeneration by upregulating the expression of bone matrix proteins and osteogenic transcription factors in bone marrow mesenchymal stem cells (BMSCs).^10^, ^17^, ^18^ These reports highlighted that SATB2 serves as a candidate bio‐factor for bone regeneration engineering. In order to further gain insights into the molecular mechanism through which SATB2 regulates osteo/odontogenic differentiation in odontogenic MSCs, we have recently conducted a comprehensive transcriptomic analysis of SATB2‐regulated expression and demonstrated the SATB2 effectively regulates numerous osteogenic regulators and marker genes in a panel of human dental stem cells, including periodontal ligament stem cells, dental pulp stem cells and stem cells from human exfoliated deciduous teeth.^19^ While these findings provided important insights into possible underlying mechanism of SATB2‐regulated osteo/odontogenic differentiation, potential upstream regulators of SATB2 it remains to be fully investigated.

Bone morphogenetic protein 9 (BMP9), also called growth differentiation factor 2, has been reported to play a pivotal role in skeletal development, bone formation and stem cell differentiation.^20^ Many transcription factors and cytokines, such as Runt‐related transcription factor 2 (*Runx2*)^21^ and Osterix (*Osx*),^22^ mediate *Bmp9*‐induced osteogenic differentiation of MSCs. We and others have recently demonstrated that BMP9 regulates dentinogenesis,^23^ tooth root development and alveolar ridge height.^24^, ^25^ Interestingly, mice with *Satb2* deficiency exhibit phenotypes similar to *Bmp* loss‐of‐function phenotypes.^26^, ^27^ Furthermore, *Satb2* is considered a downstream effector of *Bmp* signalling and directly binds to *Smad1/5* during osteoblast differentiation in facial skeletal development.^8^ Nonetheless, the detailed mechanism through which SATB2 may mediate BMP9‐induced osteo/odontogenic differentiation of odontogenic MSCs remains to be fully elucidated.

In this study, we sought to investigate the function and possible mechanism of *Satb2* inducing osteo/odontogenic differentiation of mouse incisor MSCs, as well as its potential synergy with the osteo/odontogenic signalling factor, *Bmp9*. We found that *Satb2* is expressed in mesenchymal cells in the apical papilla of mouse incisors and effectively enhances the self‐renewal capacity of mouse incisor MSCs. We further demonstrated that *Satb2* acts synergistically with *Bmp9* in inducing osteo/odontogenic differentiation of mouse incisor MSCs in vitro and in vivo. Therefore, our findings strongly suggest that a thorough understanding of *Satb2* functions should significantly facilitate our efforts to develop efficacious bone and tooth engineering approaches.

## 2 MATERIALS AND METHODS

### 2.1 Cell culture and chemicals

The 293pTP cell line was used for adenovirus packaging and amplification as previously described.^6^ Both primary mouse incisor MSCs (see below) and 293pTP cells were maintained in complete Dulbecco's modified Eagle's medium containing 10% foetal bovine serum (FBS, HyClone, New York, USA), 100 units of penicillin and 100 µg of streptomycin at 37°C in 5% CO_2_. Unless mentioned otherwise, all chemicals were purchased from Beyotime (Shanghai, China) or HyClone（New York, USA).

### 2.2 Immunofluorescence staining of *Satb2* and *Bmp9* expression in mouse incisors

The use and care of C57BL/6 mice and Sprague‐Dawley (SD) rats in this study followed an animal protocol approved by the Research Ethics Committee of College of Stomatology, Chongqing Medical University, Chongqing, China (CQHS‐REC‐2020（LSNo.55)）. Mandibular specimens from C57BL/6 mice (4‐week‐old males and females) were extracted for isolation of the incisor apical papilla. Immunofluorescence and haematoxylin and eosin (H&E) staining were carried out. Anti‐Satb2 (1:600, Abcam, Cambridge, UK) or anti‐Bmp9 (1:100; ThermoFisher Scientific, Waltham, USA) antibody and Alexa Fluor® 488‐AffiniPure goat anti‐rabbit IgG (H + L) were used as the primary and secondary antibodies, respectively. Control IgGs and an absence of primary antibody were used as negative controls.

### 2.3 Isolation, culture and characterization of mouse incisor MSCs

Primary mouse incisor MSCs were isolated from mouse incisor apical papilla tissues obtained from C57BL/6 mice (4 weeks old, both male and female) as previously reported.^28^, ^29^ See Supporting Information for details.

### 2.4 Construction and amplification of recombinant adenoviruses expressing *Bmp9*, *Satb2*, si*Satb2* and *G/Rfp*

Ad‐*Bmp9*, Ad‐*Satb2*, Ad‐*Gfp* and Ad‐*Rfp* recombinant adenoviruses were constructed using AdEasy technology as previously described.^6^, ^19^ Briefly, the coding region of the mouse *Satb2* gene was PCR‐amplified and subcloned into the adenoviral shuttle vector pAdTrack‐TOX, which was subsequently used for homologous recombination with the adenoviral backbone vector in BJ5183 bacterial cells. The resulting recombinant plasmid was verified and used for adenovirus packaging in 293pTP cells, leading to production of the adenoviral vector Ad‐*Satb2*, which also co‐expressed the marker *Gfp* for tracking infection efficiency (Figure 3Aa).

To generate siRNA adenovirus targeting mouse/rat *Satb2*, we employed the siDESIGN program (Dharmacon, a Horizon Discovery Group company, Lafayette, Colorado, USA) and designed three siRNAs that target the coding region of mouse *Satb2*, while two of these three siRNAs also target the coding region of rat *Satb2* (Table S1; Figure 3Ab). siRNA oligo cassettes were assembled into an adenoviral shuttle vector using the recently reported FAMSi system^30^ and subsequently cloned into the adenoviral backbone vector, resulting in Ad‐si*Satb2*. Recombinant adenovirus Ad‐si*Satb2* was generated in 293pTP cells. The Ad‐si*Satb2* virus also co‐expresses the marker *Rfp* for tracking infection efficiency. All constructs involving the use of PCR‐amplified fragments and oligo cassettes were verified by DNA sequencing. An analogous adenovirus Ad‐*G/Rfp* expressing both *Gfp* and *Rfp* was used as a mock control virus. Polybrene (10 µg/mL; Solarbio, Beijing, China) was used to enhance viral infection efficiency in all adenoviral infections. All cloning details and information about the reported vectors are available upon request.

### 2.5 RNA isolation, reverse transcription and quantitative RT‐PCR

Total RNA was isolated and subjected to reverse transcription using a cDNA Reverse Transcription Kit (TaKaRa Bio Inc, Shiga, Japan). See Supporting Information for details.

### 2.6 Western blot analysis

Cells were lysed, denatured, separated and transferred. The following three primary antibodies were used: anti‐SATB2 antibody (1:1000; Abcam, Cambridge, UK), anti‐BMP9 antibody (1:1000; ThermoFisher Scientific, Waltham, USA) and anti‐GAPDH antibody (1:3000; Zen Bioscience, Chengdu, China). Chemiluminescence (Beyotime, Shanghai, China) reagent was used to visualize the presence of the proteins of interest. Image J software was employed for protein band quantification.

### 2.7 Osteogenic differentiation of mouse incisor MSCs (ALP assays and alizarin red S mineralization staining)

The mouse incisor MSCs were seeded at a density of 1 × 10^5^ cells/well in 24‐well cell culture plates and infected with Ad‐*G/Rfp*, Ad‐*Satb2*, Ad‐si*Satb2*, Ad‐*Bmp9*, Ad‐*Satb2* + Ad‐*Bmp9* or Ad‐si*Satb2* + Ad‐*Bmp9*. See Supporting Information for details.

### 2.8 CCK‐8 assay

Cell proliferation was analysed using Cell Counting Kit‐8 (CCK‐8; Bioss, Beijing, China). See Supporting Information for details.

### 2.9 Crystal violet assay

Subconfluent cells were seeded in 35‐mm dishes and infected with Ad‐*Satb2*, Ad‐si*Satb2* or Ad‐*G/Rfp*. The infected cells were stained with crystal violet at the indicated time points. Macrographic staining images were recorded. The stained cells were dissolved in 10% acetic acid at room temperature, and the optical density was measured at 570‐590 nm for quantitative measurement.

### 2.10 GelMA hydrogel synthesis and preparation

Gelatin methacryloyl (GelMA) hydrogel was synthesized (see Supporting Information for details).

### 2.11 Proliferation and viability of mouse incisor MSCs encapsulated in GelMA hydrogel

The proliferation and viability of mouse incisor MSCs encapsulated in GelMA hydrogel were evaluated via CCK‐8 assays and live/dead staining. See Supporting Information for details.

### 2.12 Rat calvarial defect repair model and micro‐CT analysis

SD rats (8 weeks old) were anaesthetized, and a 5‐mm‐diameter critical‐sized defect was created on each side of the rat calvarial bone using a dental bur (Figure 6Fa). See Supporting Information for details.

### 2.13 Histologic evaluation

The retrieved rat cranial and mouse mandibular specimens were fixed, decalcified, dehydrated and paraffin embedded. Three randomly selected cross sections from each implant were stained with H&E. Trichrome staining was also carried out on rat cranial specimens. Macrographic images were documented under a bright‐field microscope (YC.YX‐2050, Japan).

### 2.14 Statistical analysis

All quantitative data are presented as the means ± SDs. The continuous data were normally distributed, and the statistical significance was determined using Student's *t* test or one‐way analysis of variance. *P* < .05 was accepted as statistically significant. All statistical analyses were conducted with the SPSS 26.0 statistical software (SPSS Inc, Chicago, USA).

## 3 RESULTS

### 3.1 *Satb2* and *Bmp9* expression pattern in mouse incisors

**FIGURE 1:**
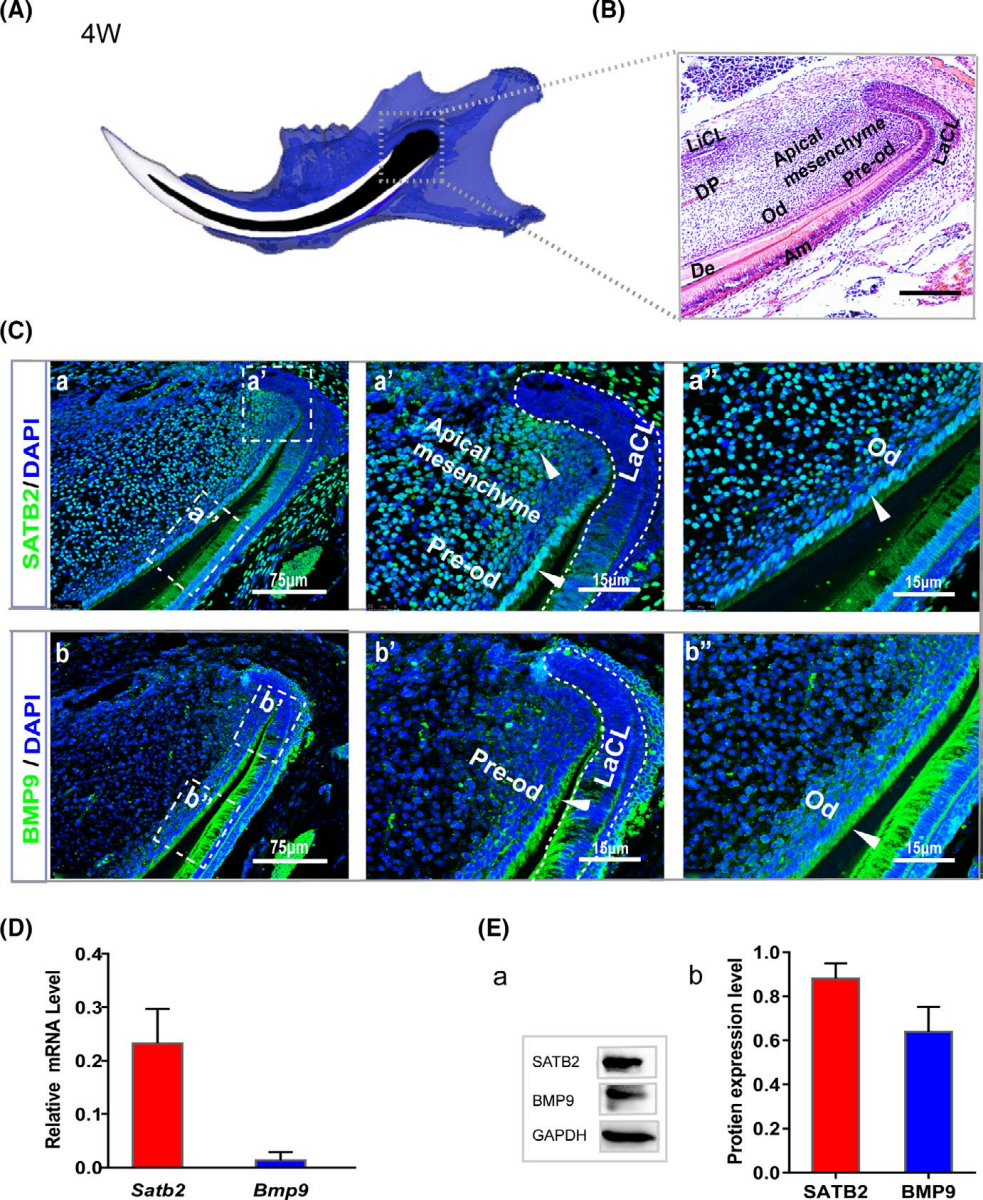
*Satb2* and *Bmp9* expression patterns in the apical region of mouse incisors. A, Schematic diagram of the mouse lower jaw and (B) H&E staining of sagittal sections from mandibular incisors of 4‐wk‐old mice showing the apical structure. Scale bar = 100 µm. Am, ameloblasts; De, dentin; LaCL, labial cervical loop; LiCL, lingual cervical loop; Od, odontoblasts; Pre‐od, pre‐odontoblasts. C, Immunofluorescence staining indicating the location of SATB2 (green) and BMP9 (green) in the apical region of incisors from 4‐wk‐old mice. Nuclei were counterstained with DAPI. (Ca,b) Scale bar = 75 µm. The boxed region in the left panel is shown as an enlarged image in the right panel. (Ca′a″b′b″) Scale bar = 15 µm. D, qPCR was used to determine the *Satb2* and *Bmp9* mRNA expression levels in the apical region of mouse incisors. Each assay condition was analysed in triplicate. E, Protein expression levels of SATB2 and BMP9 in the apical region of incisors from 4‐wk‐old mice were determined by Western blot and quantified with ImageJ. Quantitative analysis of Western blot results was performed with data from at least three independent experiments

Micro‐CT (μCT) image showed a schematic diagram of a lower jaw incisor in a 4‐week‐old mouse (Figure 1A). Magnification of the H&E‐stained image shows the apical papilla region of the incisors characterized by continuous growth. The apical mesenchyme was located between the lingual cervical loop (LiCL) and labial cervical loop (LaCL) in the mouse incisor (Figure 1B). To determine whether *Satb2* and *Bmp9* are expressed in the incisor growth region, immunofluorescence staining was conducted. *Satb2* was found to be co‐expressed with *Bmp9* in pre‐odontoblasts/odontoblasts. In addition, *Satb2* was found in the mesenchyme, where *Bmp9* expression was not detectable (Figure 1C). Furthermore, qPCR analysis and Western blot revealed the *Satb2* and *Bmp9* mRNA and protein expression levels in the incisor growth region (Figure 1D,E).

### 3.2 Isolation and characterization of stem cells in the incisor apical papilla

**FIGURE 2:**
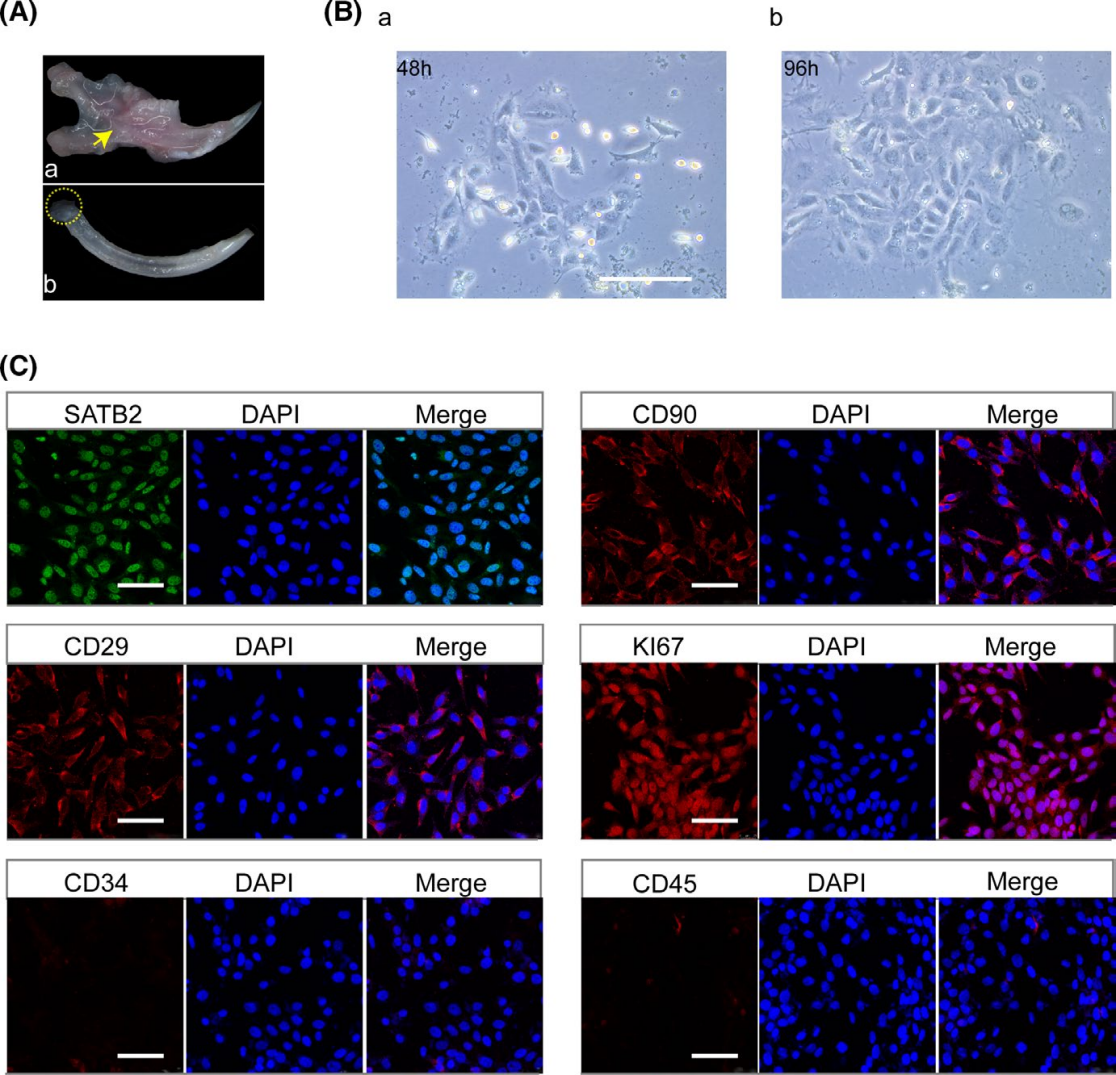
Isolation and characterization of stem cells of the incisor apical papilla. A, Isolation of mouse incisor mesenchymal stem cells (MSCs) from mouse lower incisors. The incisors of adult C57BL/6 mice were separated (a) and carefully extracted (b). The apical papilla regions (b, dotted circles) were resected and retrieved for cell isolation. B, Mouse incisor MSCs were cultured in dishes for 48 h (a) and 96 h (b). C, Endogenous expression of SATB2, the MSCs markers CD90 and CD29 and the proliferation marker KI67 in mouse incisor MSCs

To obtain insights into the role of *Satb2* in the apical mesenchyme, mouse incisor MSCs were isolated from the lower incisors of mice (Figure 2A). The primary mouse incisor MSCs grew well to at least 5 passages (Figure 2Ba‐b). Mouse incisor MSCs from passages 3‐5 were used in this study. MSCs markers (CD90, CD29) and a proliferation marker (KI67)^4^, ^5^ were expressed in the mouse incisor MSCs, while CD34 and CD45 were negative, as revealed by immunofluorescence staining. Moreover, mouse incisor MSCs were positive for SATB2 staining, which was observed in the nucleus (Figure 2C). Collectively, these results demonstrate that mouse incisor MSCs express MSCs markers and SATB2, suggesting that these cells may exhibit MSC‐like characteristics.

### 3.3 Validation of adenovirus‐mediated *Satb2* overexpression and silencing

**FIGURE 3:**
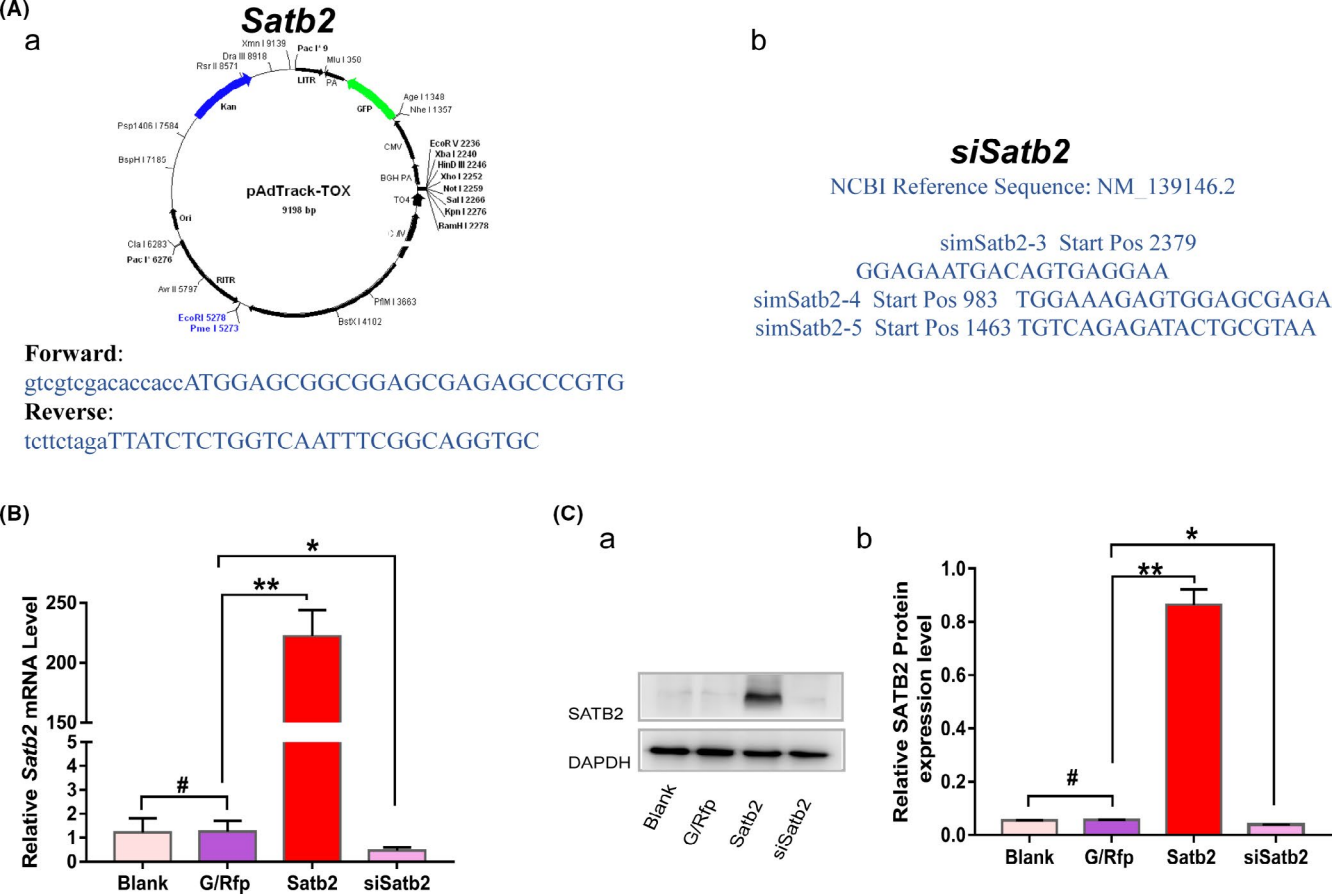
Constructs and validation of adenoviral vector‐mediated *Satb2* overexpression and silencing. Aa,b, Molecular cloning and construction of *Satb2* overexpression (a) and silencing (b) adenoviral vectors. Notably, the Ad‐si*Satb2* vector encodes three siRNAs targeting mouse *Satb2*, two of which (sim*Satb2‐4* and sim*Satb2‐5*) also target rat *Satb2* expression (NM_001109306). *Satb2* target gene and protein expression levels in mouse incisor mesenchymal stem cells (MSCs) in the Ad‐*Satb2* and Ad‐si*Satb2* groups were determined by qPCR (B) and Western blot (C). Target gene and protein expression levels in all samples were normalized to the corresponding Gapdh levels. Each assay condition was analysed in triplicate. Quantitative analysis of Western blot results was performed with data from at least three independent experiments. All values are the means ± SDs; *P <.05 and **P <.01, # no statistical significance

To investigate the function of *Satb2* in mouse incisor MSCs, we constructed recombinant adenoviruses for *Satb2* overexpression (Ad‐*Satb2*) and silencing (Ad‐si*Satb2*) (Figure 3Aa,b). We assessed the overexpression and silencing efficiency of Ad‐*Satb2* and Ad‐si*Satb2* in mouse incisor MSCs. After mouse incisor MSCs were infected with Ad‐*Satb2* or Ad‐*siSatb2*, samples were collected to obtain mRNA and protein for qPCR and Western blot analyses, respectively. *Satb2* expression at both the mRNA and protein level increased in mouse incisor MSCs after infection with Ad‐*Satb2* and decreased after infection with Ad‐si*Satb2* compared with the control and endogenous level. No significant differences were found between the blank control and Ad‐*G/Rfp* infection groups (Figure 3B,C). Thus, these results confirm that *Satb2* expression was effectively up‐ or downregulated in mouse incisor MSCs by Ad‐*Satb2* or Ad‐si*Satb2* infection, respectively.

### 3.4 *Satb2* is essential for the self‐renewal and osteo/odontogenic differentiation capabilities of mouse incisor MSCs in vitro

**FIGURE 4:**
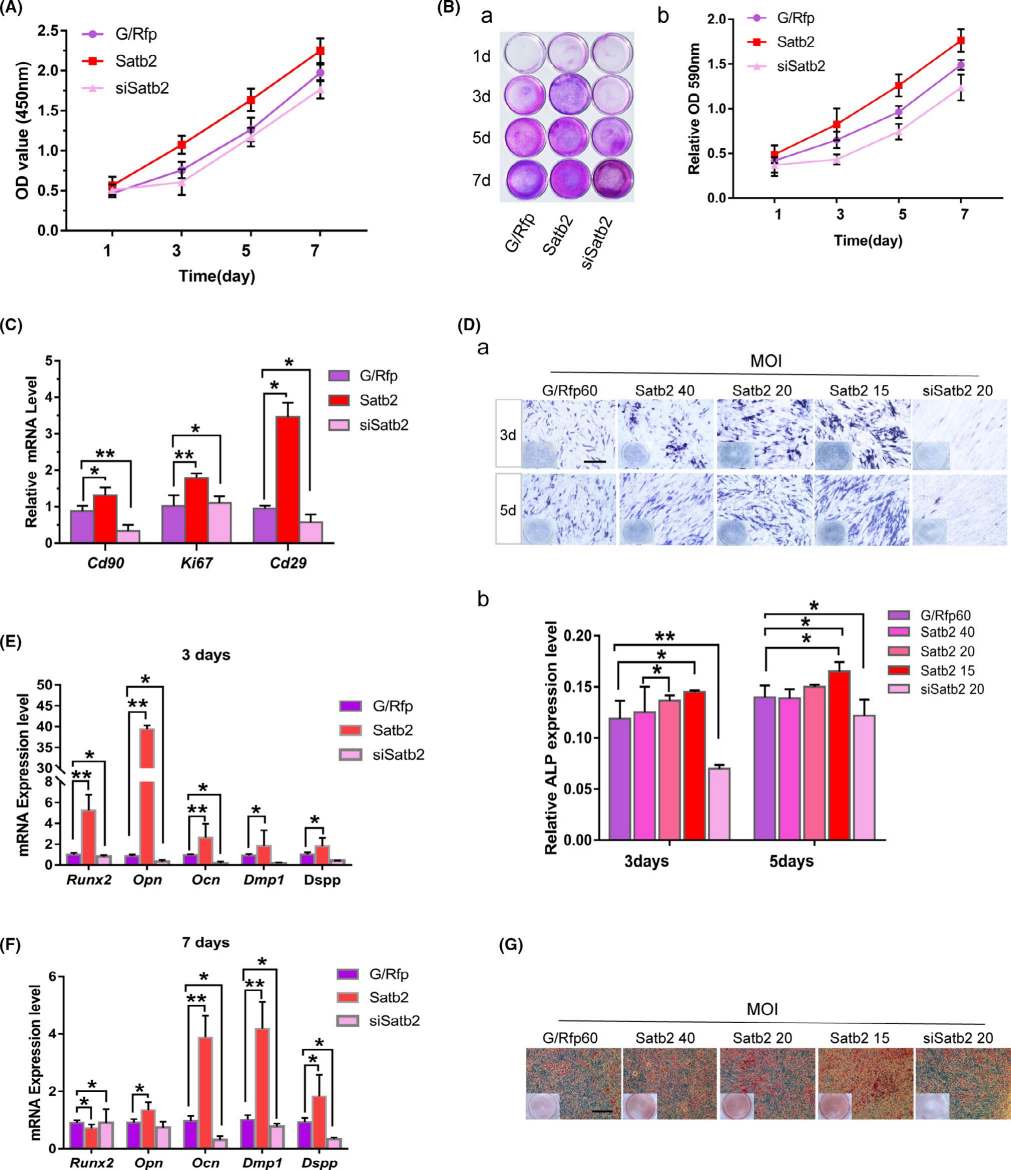
The effect of *Satb2* on the self‐renewal and osteo/odontogenic differentiation capabilities of mouse incisor mesenchymal stem cells (MSCs) in vitro. A, The proliferation of mouse incisor MSCs infected with Ad‐*G/Rfp*, Ad‐*Satb2* or Ad‐si*Satb2* and seeded at a low density was investigated with CCK‐8 assays. B, Cell proliferation was assessed via crystal violet staining. (a) Mouse incisor MSCs subjected to different treatments were plated at a low density and fixed for crystal violet staining at the indicated time points. (b) The stained cells were detached for OD measurement to quantitatively determine the 590 nm value. The assays were performed in three independent batches of experiments, and representative results are shown. C, mRNA expression levels of cell stemness markers in mouse incisor MSCs subjected to different gene modifications. D, ALP staining (a) and activity (b) were observed on days 3 and 5. Scale bar = 400 µm. E,F, Expression levels of osteo/odontogenic genes in different treatment groups were determined by qPCR on 3 and 7 d. The assays were performed in three independent batches of experiments. Representative results are shown. All values are the means ± SDs; *P <.05 and **P <.01. G, Alizarin Red S staining on day 14. Scale bar = 400 µm

To determine whether *Satb2* affects the self‐renewal properties of mouse incisor MSCs, in vitro overexpression and knockdown assays were performed with Ad‐*Satb2*‐ and Ad‐si*Satb2*‐infected mouse incisor MSCs. CCK‐8 cell proliferation assays indicated that mouse incisor MSCs infected with Ad‐*Satb2* exhibited increased proliferation compared with mouse incisor MSCs infected with Ad‐si*Satb2* or Ad‐*G/Rfp*, especially after 2 days (Figure 4A). Similarly, crystal violet staining indicated that mouse incisor MSCs infected with Ad‐*Satb2* reached confluence earlier than those infected with Ad‐si*Satb2* or Ad‐*G/Rfp* after initial seeding at the same density (Figure 4Ba). Quantitative assessment of staining confirmed that *Satb2*‐overexpressing cells had significantly higher OD values than cells in the other groups (Figure 4Bb). In addition, the proliferation of mouse incisor MSCs was inhibited after transduction with Ad‐si*Satb2*. These results indicate that *Satb2* promotes mouse incisor MSCs proliferation and enhances MSCs self‐renewal capacity. *Cd90* and *Cd29* are markers of MSC self‐renewal. The *Cd90*, *Cd29* and *Ki67* mRNA levels were increased by Ad‐*Satb2* infection but reduced by Ad‐si*Satb2* infection (Figure 4C). These data indicate that *Satb2* may enhance the self‐renewal properties of mouse incisor MSCs.

We next evaluated the role of *Satb2* in mouse incisor MSCs osteo/odontogenic differentiation. Cells infected with Ad‐*Satb2* exhibited higher ALP activity on day 3 and 5 after infection than cells in the other groups (Figure 4Da,b), and the effect was dependent on the Ad‐*Satb2* virus dose. Next, we determined the expression levels of the osteoblast transcription factors *Runx2*, *Opn* and *Ocn*. Ad‐*Satb2* infection was shown to significantly induce the expression of *Runx2* and *Opn* but slightly increase *Ocn* on 3 days. The expression of *Runx2* and *Opn* was fell, while *Ocn* was risen remarkably on 7 days. Silence the expression of *Satb2* in dental MSCs suppressed *Runx2, Opn* and *Ocn* in two time points (Figure 4E,F). These results are consistent with the previous observation that *Satb2* regulates osteo/odontogenic differentiation transcription factors in mouse BMSCs.^31^ Moreover, ARS staining demonstrated that *Satb2* significantly enhanced mineralized node formation (Figure 4G).

As important markers of odontoblastic differentiation, the expression of dentin matrix protein 1 (*Dmp1*) and dentin sialophosphoprotein (*Dspp*) in mouse incisor MSCs was also assessed. We found *Satb2* overexpression infected with Ad‐*Satb2* slightly promoted *Dmp1* and *Dspp* expression on day 3 and markedly upregulated on day 7, which was inhibited by knockdown of *Satb2* expression with Ad‐si*Satb2* infection (Figure 4E,F). Thus, these results indicate that *Satb2* can induce the odontoblast‐like differentiation process in mouse incisor MSCs.

### 3.5 *Satb2* participates in *Bmp9*‐induced osteo/odontogenic differentiation of mouse incisor MSCs in vitro

**FIGURE 5:**
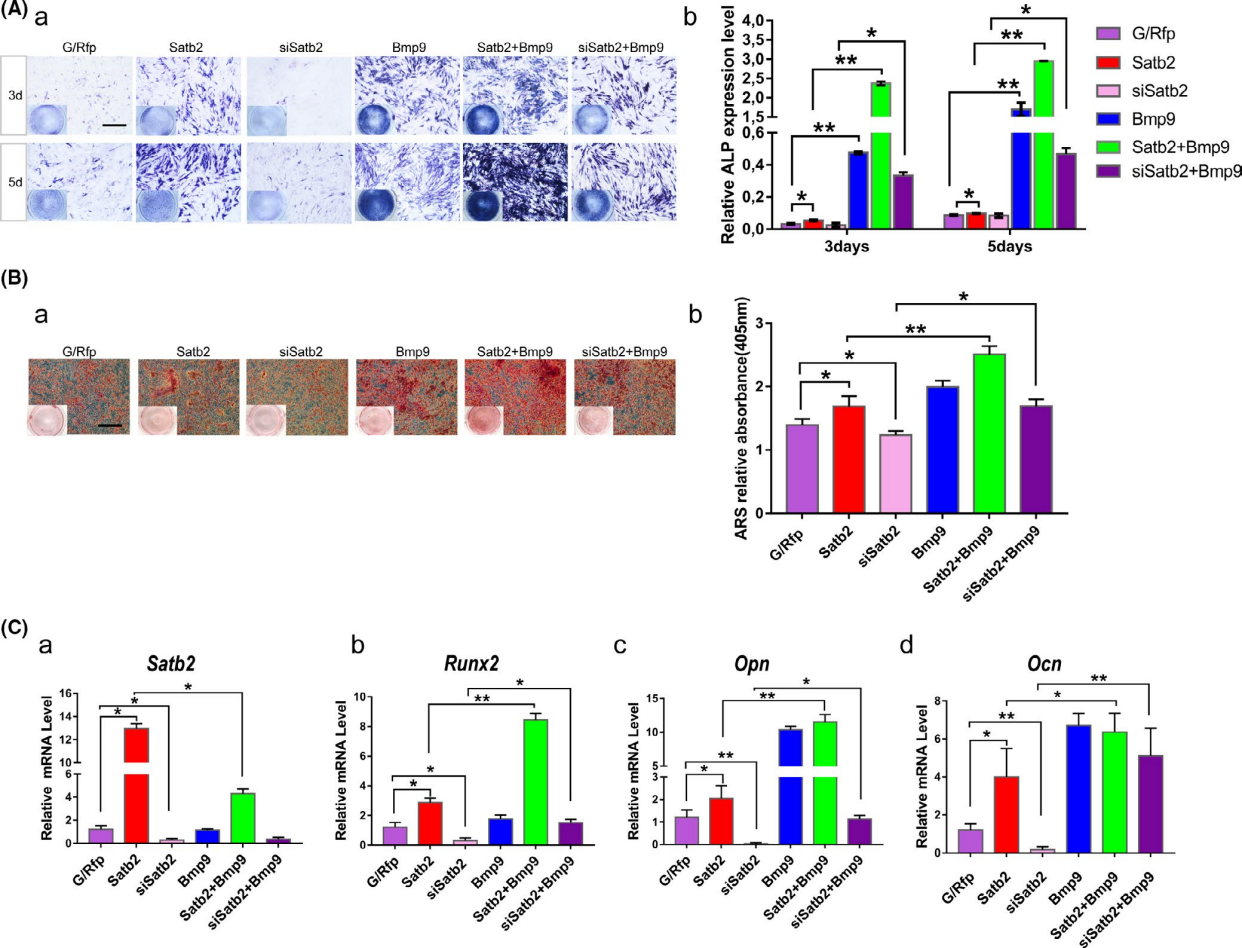
*Satb2* participates in *Bmp9*‐induced osteo/odontogenic differentiation of mouse incisor MSCs in vitro. A, Cells were seeded in 24‐well culture plates and infected with Ad‐*G/Rfp*, Ad‐*Satb2*, Ad‐si*Satb2*, Ad‐*Bmp9*, Ad‐*Satb2* + Ad‐*Bmp9* or Ad‐si*Satb2* + Ad‐*Bmp9* for 3 and 5 d. The effects of *Satb2* modulation on ALP staining (a) and ALP activity (b) in *Bmp9*‐induced mouse incisor MSCs were assessed. Scale bar = 400 µm. B, Alizarin Red S staining (a) and quantification (b). Infected mouse incisor MSCs were maintained in matrix mineralization culture medium for 14 d. Matrix mineralization nodules were stained with Alizarin Red S. Stained cells were imaged under a microscope. Scale bar = 400 µm. Ca‐d, *Satb2*, *Runx2*, *Opn* and *Ocn* mRNA levels were determined in different groups after 3 d. All values are the means ± SDs; *P <.05 and **P <.01

*Satb2* was co‐expressed with *Bmp9* in pre‐odontoblasts/odontoblasts. To assess whether *Satb2* and *Bmp9* exhibit crosstalk during osteo/odontogenic differentiation, we analysed the effect of *Satb2* on *Bmp9*‐induced osteoblast differentiation of mouse incisor MSCs in vitro. After mouse incisor MSCs were infected with Ad‐*Satb2* or Ad‐si*Satb2* separately, *Bmp9*‐induced osteo/odontogenic was detected via ALP and ARS staining, followed by quantitative analysis (Figure 5A,B). Compared with transduction with only Ad‐*Satb2* or Ad‐*Bmp9*, co‐transduction with Ad‐*Satb2* and Ad‐*Bmp9* strongly augmented ALP activity (Figure 5Aa,b). Interestingly, *Satb2* mRNA expression was not induced after Ad‐*Bmp9* infection, suggesting that *Satb2* may not act as a direct target of *Bmp9* osteogenic signalling (Figure 5Ca). Nonetheless, we found that *Runx2*, *Ocn* and *Opn* expression was upregulated by *Satb2* in *Bmp9*‐induced osteo/odontogenic differentiation (Figure 5Cb‐d). ARS staining and quantitative analysis confirmed this tendency in the late stage of mineralization (Figure 5Ba,b).

### 3.6 Enhancement of calvarial bone healing in rats synergistically induced by *Satb2* and *Bmp9*

**FIGURE 6:**
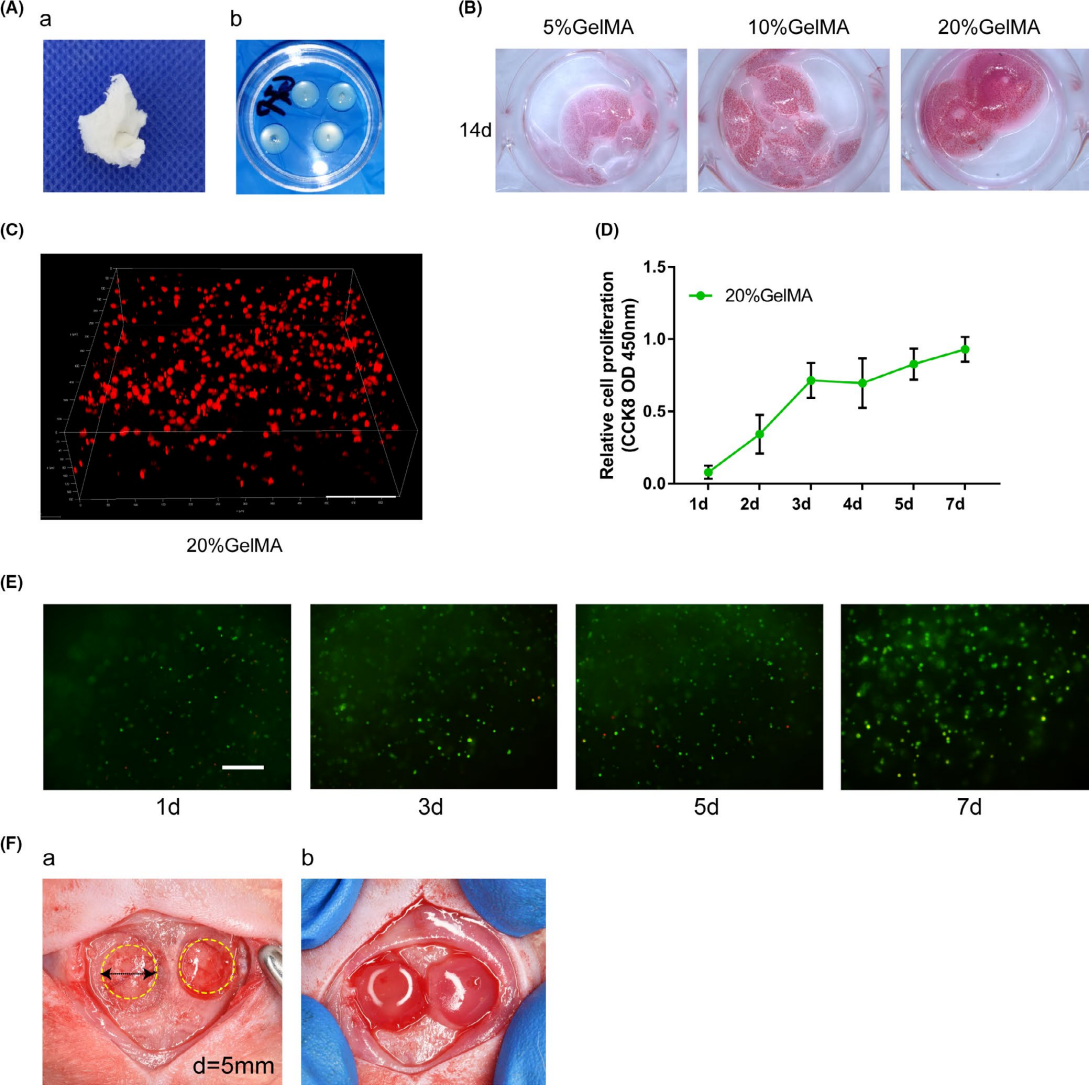
Synthesis and preparation of GelMA hydrogels for use as mouse incisor MSCs scaffolds. A, Dry GelMA (a) and GelMA hydrogel formation (b). B, Cells cultured with GelMA hydrogels at different concentrations for 14 d. C, Representative image of the encapsulated mouse incisor mesenchymal stem cells in 20% (w/v) GelMA hydrogel at 24 h. Scale bar = 100 µm. D, Cell growth in 20% (w/v) GelMA hydrogels was assessed with CCK‐8 assays. E, Live/dead staining of cells in 20% (w/v) GelMA hydrogels after 24 h of encapsulation. Live cells (green), dead cells (red). Scale bar = 400 µm. F, Five‐millimetre‐diameter defects were drilled into each side of each rat calvaria (Fa), and GelMA hydrogels with modified cells were implanted (Fb)

The concentration of GelMA hydrogel suitable for supporting in vitro mouse incisor MSCs survival and proliferation was assessed. On day 14, 5% and 10% (w/v) GelMA hydrogels began to degrade (Figure 6B). However, 20% (w/v) GelMA hydrogel retained a stiff, self‐standing microporous environment and promoted cell adhesion, proliferation and rapid 3D seeding. Thus, 20% (w/v) GelMA hydrogel was used in subsequent in vitro and in vivo studies. Mouse incisor MSCs (5 × 10^6^) were suspended in 50 µL of 20% (w/v) GelMA hydrogel and allowed to form into a hemispheric 3D shape (Figure 6Ab). Then, via confocal laser scanning microscopy, we observed that mouse incisor MSCs were uniformly distributed throughout the 3D GelMA hydrogel at 24 hours (Figure 6C). CCK‐8 assays and live/dead staining were applied to detect mouse incisor MSCs proliferation and viability in the GelMA hydrogel. As shown in Figure 6D,E, mouse incisor MSCs were maintained and exhibited high proliferation and viability up to day 7. These results demonstrated that hemispheric 3D GelMA hydrogel at a 20% (w/v) concentration was appropriate for subsequent in vivo studies.

**FIGURE 7:**
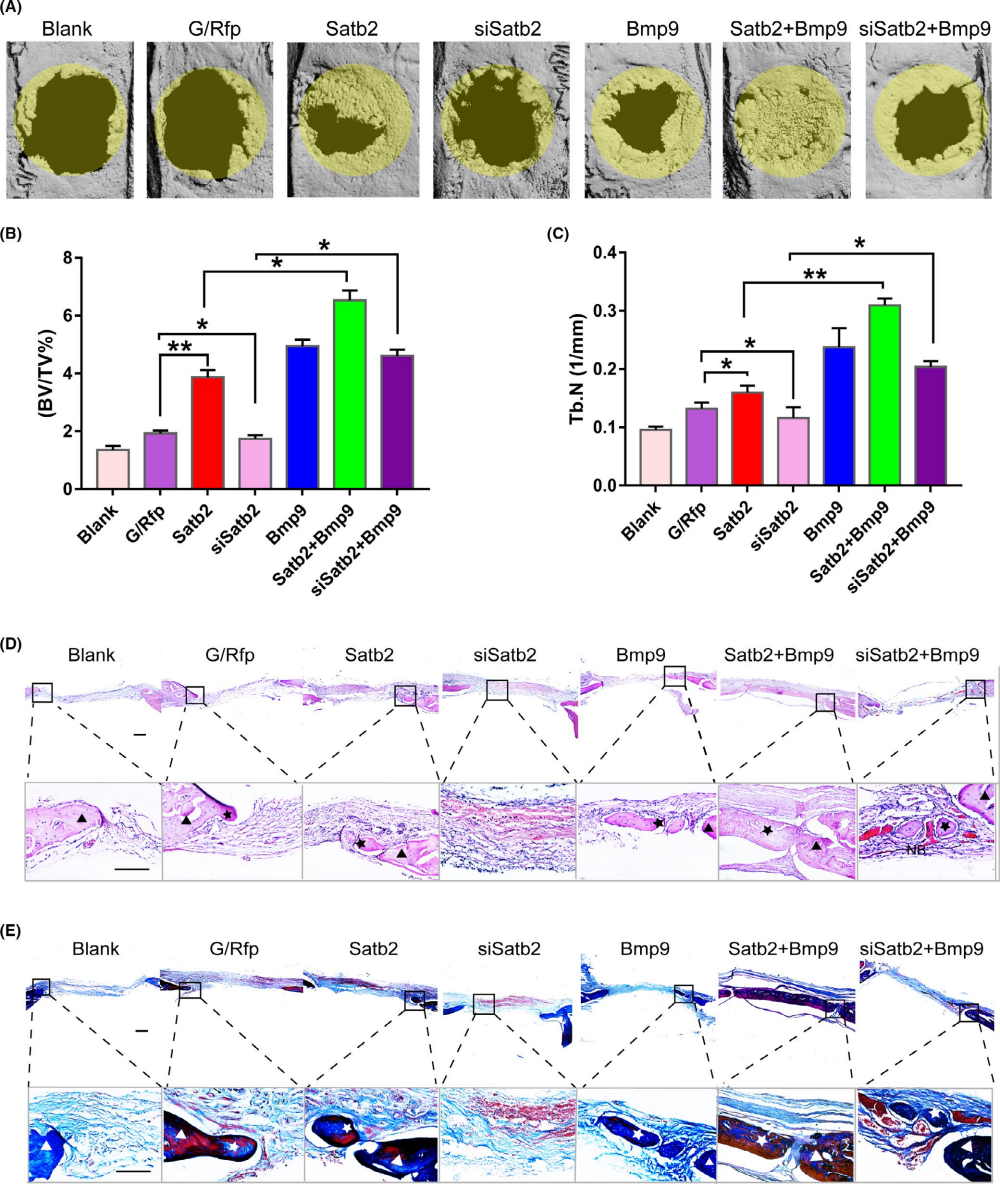
Enhancement of calvarial bone healing in rats synergistically induced by *Satb2* and *Bmp9*. A, Calvarial bone defect specimens were harvested at 10 wks post‐implantation, and 3D µCT images of the rat calvaria were reconstructed. The area inside the yellow circle is the new bone area. B,C, BV/TV and Tb.N were analysed using μCT software. All values are the means ± SDs; *P <.05 and **P <.01. D, H&E staining. E, Masson staining. Scale bar = 200 µm; ▲ indicates the host bone, and ★ indicates the newly formed bone. The magnified images show areas of new bone formation. Scale bar = 50 µm

To investigate the participation of *Satb2* in bone healing, two critically sized defects with a non‐healing full thickness diameter of 5 mm were made in both sides of rat calvaria (Figure 6Fa). The defects were filled with GelMA hydrogel embedded with genetically modified mouse incisor MSCs (Figure 6Fb). To evaluate new bone formation, μCT images were acquired at 10 weeks and were then analysed. Representative results of new bone formation are shown in Figure 7A. Quantitative analysis of the µCT images indicated that the volume (bone volume fraction, BV/TV) and quantity (number of trabeculae, Tb.N) of new bony tissue were increased by *Satb2*. In contrast, silencing *Satb2* expression in mouse incisor MSCs significantly decreased BV/TV and Tb.N (Figure 7B,C). Furthermore, robust bony masses were retrieved from defects seeded with cells infected with both Ad‐*Satb2* and Ad‐*Bmp9*. Interestingly, compared with bony tissue formed by mouse incisor MSCs infected with Ad‐*Satb2* or Ad‐*Bmp9* alone, bony tissue formed by mouse incisor MSCs infected with both Ad‐*Satb2* and Ad‐*Bmp9* exhibited a significantly increased BV/TV and Tb.N. Moreover, *Bmp9* at least partially reversed the inhibitory effect of transduction with si*Satb2* on new bone formation.

H&E staining results revealed that mouse incisor MSCs infected with Ad‐*Satb2* or Ad‐*Bmp9* separately formed evident trabecular bone. However, trabecular bone formation was significantly increased by combined transduction of mouse incisor MSCs with *Satb2* and *Bmp9* (Figure 7D). Masson trichrome staining confirmed that mouse incisor MSCs transduced with *Satb2* or *Bmp9* separately formed obvious mature and mineralized bone matrices. However, maturity and mineralization were significantly augmented in mouse incisor MSCs transduced with both *Satb2* and *Bmp9* (Figure 7E). Collectively, these results strongly suggest that *Satb2* and *Bmp9* may act synergistically in inducing osteo/odontogenic differentiation of mouse incisor MSCs.

## 4 DISCUSSION

In this study, we first found that *Satb2* was broadly expressed in the apical mesenchyme and coactivated with *Bmp9* in pre‐odontoblasts/odontoblasts from mouse incisors. Then, we further investigated the positive regulatory effect of *Satb2* on the proliferation, self‐renewal and osteo/odontogenic differentiation properties of mouse incisor MSCs and examined its downstream target gene. *Satb2* was found to play a collaborative role in *Bmp9*‐induced osteo/odontogenic differentiation by upregulating *Runx2* and *Opn* expression. These results strongly suggest that *Satb2* plays an essential role in mouse incisor MSCs self‐renewal properties and a cooperative role in *Bmp9*‐induced osteo/odontogenic differentiation of mouse incisor MSCs.

Self‐renewal of MSCs produces new dentin in the mouse incisor.^5^ Gene overexpression and silencing experiments revealed that *Satb2* enhanced the self‐renewal capability of mouse incisor MSCs and increased CD90/Thy1 and CD29 expression in vitro. CD90/Thy1 and CD29 are archetypal membrane markers of odontogenic MSCs that increase the incisor growth rate by contributing to the formation of odontoblasts and pulp cells.^5^, ^32^ Our findings indicated that mouse incisor MSCs from incisor mesenchymal tissue exhibit stem cell properties. The broad *Satb2* expression and its positive regulation of CD90/Thy1 and CD29 expression suggest that *Satb2* may be a potent regulator of mouse incisor MSCs self‐renewal.

Dentin derived from odontoblasts comprises >70% of the entire tooth structure and functions as a protective barrier for dental pulp.^33^, ^34^ Dentin formation requires MSCs to differentiate into odontoblast‐like cells.^5^, ^32^, ^35^ Loss of dentin causes several tooth problems, such as pulpitis. External molecules that can stimulate odontoblast differentiation are urgently needed to accelerate dentin repair.^36^ *Dspp* and *Dmp1* are two important odontoblastic differentiation marker genes in mouse incisor MSCs.^37^, ^38^, ^39^, ^40^ Here, Ad‐*Satb2*‐infected cells generally exhibited greater *Dmp1* and *Dspp* expression on later stage of inducing, as well as higher ALP activity and greater formation of mineralized nodules. These results indicate that *Satb2* promotes odontoblast‐like differentiation of mouse incisor MSCs, suggesting that, indeed, the expression of *Satb2* in mouse incisor MSCs could be important for dentin morphogens and that *Satb2* might be a candidate molecule for dentin regeneration.

Mouse incisors can grow without interruption throughout the animal's lifetime and thus provide a favourable model for understanding tissue renewal and tooth engineering.^41^ Reports have indicated that a ‘stem cell pool’ forms in the apical region of the mouse incisor root, from which the cells persistently regenerate and differentiate into new tissue, thus ensuring that the incisor grows continuously.^3^, ^41^, ^42^, ^43^ *Satb2* enhanced ALP activity and calcium nodule deposition in mouse incisor MSCs. In addition, the osteo/odontogenesis‐related factors *Runx2 and Opn* tended to be upregulated after transduction with *Satb2* on 3 days. *Dspp*, *Dmp1* and *Ocn* tended to be upregulated markedly on 7 days. These results indicate that *Satb2* promotes osteo/odontogenic differentiation of mouse incisor MSCs. Similarly, deficiency of *Satb2* in bone has been shown to impair differentiation of osteoblast progenitors. Collectively, these results suggest that *Satb2*‐induced osteo/odontogenic differentiation of mouse incisor MSCs is a promising strategy for osteo/odontogenic regeneration and tooth engineering.

Osteo/odontogenic differentiation is a well‐orchestrated process and requires interactions among many factors.^44^ BMP9 is the BMP with the greatest potential to induce osteo/odontogenic differentiation in odontogenic stem cells and has been found to be useful as an efficacious bio‐factor in tooth engineering in many published studies.^6^, ^24^, ^45^ We found that *Satb2* was coactivated with *Bmp9* in pre‐odontoblasts/odontoblasts, while *Satb2* but not *Bmp9* was activated in the apical mesenchyme. Odontoblast precursors migrate from the apical mesenchyme to the odontogenic region, where they differentiate into dentin‐forming odontoblasts. To better understand the role of *Satb2* in osteo/odontogenic differentiation, *Bmp9* was introduced in this study. Our results showed that *Satb2* elevated *Bmp9*‐induced osteo/odontogenic differentiation of mouse incisor MSCs in vitro and in vivo. Additionally, *Bmp9* reversed the inhibition of osteo/odontogenic differentiation after transduction with Ad‐si*Satb2*. In this study, we found that *Runx2* and *Opn* are interactive genes in the process by which *Satb2* participates in *Bmp9*‐induced osteo/odontogenic differentiation.

*Satb2* has been reported to repair bone defects in mice^46^; thus, scaffolds seeded with Ad‐*Satb2*‐infected mouse incisor MSCs were implanted in a calvarial bone defect model. We found that *Satb2* promoted bone regeneration, accompanied by increases in BV/TV and Tb.N. Importantly, we demonstrated that compared with other groups, mouse incisor MSCs infected with Ad‐*Satb2* and Ad‐*Bmp9* significantly enhanced the healing of bone defects. Our study provides the first confirmation that combined expression of *Satb2* and *Bmp9* in stem cells is a promising gene therapy approach in bone regeneration.

Previous studies have revealed that SATB2 deletion or mutation results in phenotypes similar to those of BMP deficiency in humans and mice, such as cleft palate and calvarial defects.^26^ Furthermore, loss of BMP signalling in the apical region led to a reduction in *Satb2* expression in this region.^47^ Interestingly, Smad1/5 directly binds to the *Satb2* promoter to induce osteoblast differentiation during facial skeletal development.^8^, ^26^ Smad1/5/8 are essential for proper *Bmp9*‐induced osteogenic differentiation of MSCs.^48^ However, to date, few studies have addressed the relationship between *Satb2* and *Bmp9* in odontogenic stem cells. Reports have indicated that both *Bmp* and Hedgehog (*Hh*) signalling is indispensable for inducing *Satb2* expression in neural crest cells of zebrafish. Unexpectedly, *Bmp9* stimulation did not induce *Satb2* mRNA expression in mouse incisor MSCs. Whether *Satb2* indirectly interacts with *Bmp9* to induce osteo/odontogenic differentiation requires further study for verification.

Our previously demonstrated that SATB2 was expressed in multiple types of human odontogenic stem cells.^19^ Moreover, SATB2 was shown to regulate the expression of MSC markers and osteogenic genes. In this report, we further confirm the important osteogenic role of *Satb2* in mouse incisor MSCs. More importantly, we demonstrated *Satb2* cross‐talked with and synergized with *Bmp9* in regulating osteo/odontogenic differentiation of mouse incisor MSCs. This knowledge should facilitate further efforts on SATB2/BMP9‐induced oral progenitor‐based dental tissue engineering. In conclusion, we demonstrated that *Satb2* is broadly expressed in the apical mesenchyme in mouse incisor MSCs and that *Satb2* promotes mouse incisor MSCs self‐renewal and osteo/odontogenic differentiation by upregulating *Cd90*, *Cd29*, *Ki67*, *Ocn*, *Opn*, *Runx2*, *Dspp* and *Dmp1*. We further demonstrated that combined expression of *Satb2* and *Bmp9* is a new strategy to induce osteo/odontogenic differentiation of mouse incisor MSCs in vitro and in vivo. Thus, our results strongly suggest that *Satb2*, in combination with *Bmp9*, could be a new efficacious bio‐factor for osteogenic regeneration and tooth engineering.

## CONFLICT OF INTEREST

The authors declare no conflict of interest.

## AUTHOR CONTRIBUTIONS

Qiuman Chen contributed to the study design, data acquisition, analysis, and interpretation, and drafting of the manuscript; Liwen Zheng and Yuxin Zhang contributed to the data acquisition and analysis and critical revision of the manuscript; Xia Huang and Feilong Wang contributed to the data acquisition and analysis and critically revised the manuscript; Shuang Li, Zhuohui Yang, Fang Liang, Jing Hu, Yucan Jiang, Yeming Li and Pengfei Zhou contributed to the data acquisition and analysis and drafted the manuscript; Wenping Luo and Hongmei Zhang contributed to the study conception and design and data interpretation, as well as drafted and critically revised the manuscript. All authors provided final approval and agree to be accountable for all aspects of the work.

## Supporting information

Supplementary Material

## Data Availability

The data that support the findings of this study are available from the corresponding author upon reasonable request.
