# Supplementary material for: Special AT‐rich sequence‐binding protein 2 (Satb2) synergizes with Bmp9 and is essential for osteo/odontogenic differentiation of mouse incisor mesenchymal stem cells
Source: Cell Prolif. 2021 Mar 4;54(4):e13016. doi: 10.1111/cpr.13016 (PMC8016638; doi:10.1111/cpr.13016)
Supplement: Supplementary file 1 — Supplementary Material [file CPR-54-e13016-s001.docx]

Special AT-rich Sequence-binding Protein 2 (*Satb2*) Synergizes with *Bmp9* and is Essential for Osteo/odontogenic Differentiation of Mouse Incisor Mesenchymal Stem Cells

**Running title**: ***Satb2* Potentiates *Bmp9*-induced** **Osteo/****odontogenesis**

Qiuman Chen^1,2^, Liwen Zheng^1,2^, Yuxin Zhang^1,2^, Xia Huang^1,3^, FeilongWang^1,2^, Shuang Li^1,2^, Zhuohui Yang^1,2^, Fang Liang^1,2^, Jing Hu^1,2^, Yucan Jiang^1,2^, Yeming Li^1,2^, Pengfei Zhou^1,3^, Wenping Luo^1,3^*, Hongmei Zhang^1,2^*

^1^Chongqing Key Laboratory for Oral Diseases and Biomedical Sciences, the Affiliated Hospital of Stomatology of Chongqing Medical University, Chongqing, China
^2^Department of Pediatric Dentistry, the Affiliated Stomatology Hospital, Chongqing Medical University, Chongqing, China
^3^Chongqing Municipal Key Laboratory of Oral Biomedical Engineering of Higher Education, Chongqing, China
* Corresponding authors

**Correspondence:**Hongmei Zhang, DDS, PhD

Chongqing Key Laboratory for Oral Diseases and Biomedical Sciences

The Affiliated Hospital of Stomatology

Chongqing Medical University

426 Songshibei Road, Chongqing 401147, China
Email: hmzhang@hospital.cqmu.edu.cn

Wenping Luo, DVM, PhD

Chongqing Key Laboratory for Oral Diseases and Biomedical Sciences

The Affiliated Hospital of Stomatology

Chongqing Medical University

426 Songshibei Road, Chongqing 401147, China
Email: wenpingl@hospital.cqmu.edu.cn

**SUPPLEMENTAL MATERIALS AND METHODS**

***Isolation, Culture and Characterization of mouse incisor MSCs***

Briefly, the apical papilla tissue was exposed after the mandibular bone was removed and then treated with 2% collagenase to separate the mesenchyme from the surrounding epithelium. The mesenchymal tissue was minced into small tissue bits, digested with type Ι collagenase, and plated in tissue culture plates. The isolated mouse incisor MSCs were cultured with complete DMEM in 6-cm dishes and passaged every 3-5 days. All mouse incisor MSCs used in this study were subjected to fewer than five passages. The expression of stem cell markers was assessed using immunofluorescence staining with anti-CD90 antibody (1:100,ThermoFisher Scientific, Waltham，USA), anti-CD29 antibody (1:100,Bioss,Beijing,China), anti- Ki67 antibody (1:100,Bioss, Beijing, China), anti-CD34 antibody (1:100,Bioss, Beijing, China) and anti-CD45 antibody (1:100,Bioss, Beijing, China).

***RNA Isolation, Reverse Transcription and*** ***Quantitative RT-PCR***

Gene expression levels in cells and tissue were analyzed via quantitative real-time PCR (qRT−PCR). The primer sequences for the examined genes are listed in Supporting Information Table 1. The analyses were accomplished with an ABI Prism 7500 Real-Time PCR System using SYBR Green PCR master mix reagent (Bimake, Houston，USA). *Gapdh* was used as a reference gene. The relative expression values were calculated using the 2^-ΔΔCt^ method.

***Osteogenic Differentiation of Mouse Incisor MSCs (ALP Assays and Alizarin Red S Mineralization Staining)***

At 3 and 5 days after infection, alkaline phosphatase (ALP) staining and ALP activity quantification assays were conducted according to the protocol of an NBT/BCIP staining kit and Alkaline Phosphatase Assay Kit (Beyotime, Shanghai, China).

For the matrix mineralization assay, infected mouse incisor MSCs were cultured in the presence of ascorbic acid (50 mg/mL) and β-glycerophosphate (10 mM) for 14 days. Mineralized matrix nodules were stained with Alizarin Red S (ARS). Briefly, after being fixed and washed, the cells were incubated with 1% ARS (pH 4.0-6.0, Sigma-Aldrich，St. Louis, MO USA) for 10 min and thoroughly rinsed with tap water. The staining of calcium mineral deposits (red) was recorded under bright field microscopy. For quantitative analysis, ARS staining in cells was dissolved with 10% cetylpyridinium chloride monohydrate (Solarbio, Beijing China), and the absorbance at 405 nm was measured using a microplate reader (Perkin Elmer, Waltham, USA). Each experimental condition was prepared in triplicate.

***CCK-8 Assay***

Cells were infected with Ad-*Satb2*, Ad-si*Satb2* or Ad-*G/Rfp* and plated on 96-well cell culture plates at 3×10^3^ cells per well. At 24 h after infection, 100 μl of cell culture medium containing 10 μl CCK-8 reagent was added to each well, and the plate was incubated at 37°C for 2 h. The absorbance was measured at 450 nm in a microplate reader (Perkin Elmer, Waltham, USA). All experiments were carried out in triplicate.

***GelMA*** ***Hydrogel Synthesis and Preparation***

Briefly, 10 g gelatin powder (Solarbio, Beijing, China) was added to 100 ml PBS solution and stirred vigorously at 60°C for 1 h until fully dissolved. Subsequently, 8 ml methacrylic anhydride (Sigma, St. Louis, MO USA) was added to this solution and stirred at 45°C for 2.5 h. The mixture was dialyzed in deionized water through dialysis tubing with a 12-14 kDa molecular weight cutoff (Solarbio, Beijing, China) for 5 to 7 days at 37°C to completely remove the cytotoxic, low-molecular-weight impurities, including unreacted methacrylic anhydride and methacrylic acid byproducts. The water was changed every day to remove the methacrylic acid. Subsequently, the solution was sterilized through 0.22-µm filters, lyophilized for one week and then stored at -80°C for future use.

The lyophilized GelMA (5%, 10% and 20% w/v) (Figure 6Aa) was fully dissolved in DMEM/high glucose. Then, 0.05% w/t photoinitiator (PI, Irgacure2959; Sigma-Aldrich，St. Louis, MO USA) was added to GelMA solution and incubated at 50°C until it was fully dissolved so it could be subsequently used for cell encapsulation.

For cell encapsulation, transduced mouse incisor MSCs were trypsinized and resuspended in liquid GelMA and 0.05% w/v PI at a concentration of 1×10^6^ cells/ml, followed by exposure to 2.9 mW/cm^2^ UV light (365 nm) for 50 seconds to form a hydrogel on 35-mm dishes (Figure 6Ab) and incubation under standard culture conditions. The culture medium was changed every 3 days.

***Proliferation and Viability of Mouse Incisor MSCs Encapsulated in GelMA Hydrogel***

CCK-8 assays were conducted as described above. For live/dead staining, 2 mM calcein-AM and 1.5 mM PI were added to the cell-laden hydrogels in Assay Buffer (Solarbio, Beijing, China) for 15–20 min. Detection was performed on days 1, 3, 5, and 7. The samples were then imaged using an EVOS FL Auto Imaging System (ThermoFisher Scientific, Waltham, USA).

***Rat Calvarial Defect Repair Model and Micro-CT Analysis***

The rats with defects were randomly divided into 7 groups to receive the following implants: (1) GelMA hydrogel alone (Control group); (2) Ad-G/*R**fp*+ GelMA hydrogel; (3) Ad-*Satb2*+GelMA hydrogel; (4) Ad-*siSatb2*+GelMA hydrogel; (5) Ad-*Bmp9*+GelMA hydrogel; (6) Ad-*Satb2*+Ad-*Bmp9*+GelMA hydrogel; and (7) Ad-si*Satb2*+Ad-*Bmp9*+GelMA hydrogel.

At 10 weeks after surgery, the healing status of calvarial defects was monitored via radiographic examination. At the endpoint, all retrieved specimens were fixed and scanned via μCT (Viva CT 40, Scanco Medical, Bassersdorf, Switzerland). The results were quantitatively analyzed by calculating the bone volume fraction (BV/TV) and trabeculae of newborn bone (Tb. N) in defect areas with μCT software.

**Supplemental Table 1.** Table 1List of the primers used in the study

| Gene | Sequence | Use |
| --- | --- | --- |
| *Gapdh* | Forward ACCCAGAAGACTGTGGATGG | qPCR |
|  | Reverse CACATTGGGGGTAGGAACAC |  |
| *Satb2* | Forward CTTCCTCAACCTGCCTGAAG |  |
|  | Reverse GTTGTCGGTGTCGAGGTTTT |  |
| *Cd29* | Forward TGGACAATGTCACCTGGAAA |  |
|  | Reverse TGTGCCCACTGCTGACTTAG |  |
| *Cd90* | Forward ACTGCCGCCATGAGAATAAC |  |
|  | Reverse ATCCTTGGTGGTGAAGTTGG |  |
| *Ki67* | Forward CAGTACTCGGAATGCAGCAA |  |
|  | Reverse CAGTCTTCAGGGGCTCTGTC |  |
| *Ocn* | Forward ACAGTCTGTGGCAGCTGATG |  |
|  | Reverse CACGGAAACGCTCTAGGAAG |  |
| *Opn* | Forward TCTGATGAGACCGTCACTGC |  |
|  | Reverse AGGTCCTCATCTGTGGCATC |  |
| *Runx2* | Forward CCCAGCCACCTTTACCTACA |  |
|  | Reverse TATGGAGTGCTGCTGGTCTG |  |
| *Satb2* | gtcgtcgacaccaccATGGAGCGGCGGAGCGAGAGCCCGTG | cloning |
|  | tcttctagaTTATCTCTGGTCAATTTCGGCAGGTGC |  |
| *simSatb2-3* | aattaaaaaGGAGAATGACAGTGAGGAAttttt | siRNA silencing |
|  | agctaaaaaTTCCTCACTGTCATTCTCCttttt |  |
| *simSatb2-4* | aattaaaaaTGGAAAGAGTGGAGCGAGAttttt |  |
|  | agctaaaaaTCTCGCTCCACTCTTTCCAttttt |  |
| *simSatb2-5* | aattaaaaaTGTCAGAGATACTGCGTAAttttt |  |
|  | agctaaaaaTTACGCAGTATCTCTGACAttttt |  |
